# The High Prevalence of High Normal Blood Pressure and the Differential Association of Grip Strength Metrics with Hypertension in a National Sample of Chinese College Students

**DOI:** 10.3390/jcm15030992

**Published:** 2026-01-26

**Authors:** Yang Yang, Ziyue Sun, Shan Cai, Jiajia Dang, Yunfei Liu, Jiaxin Li, Tianyu Huang, Ruolan Yang, Jinghong Liang, Peijin Hu, Jun Ma, Zhixin Zhang, Yi Song

**Affiliations:** 1 Institute of Child and Adolescent Health, School of Public Health, Peking University, Beijing 100191, China; 2516395133@bjmu.edu.cn (Y.Y.); sunziyue@bjmu.edu.cn (Z.S.); dangjj@bjmu.edu.cn (J.D.); liuyunfei_pku@163.com (Y.L.); lijiaxin@bjmu.edu.cn (J.L.); tyhuang@pku.edu.cn (T.H.); 2010306116@stu.pku.edu.cn (R.Y.); 2516392194@bjmu.edu.cn (J.L.); peijinhu@bjmu.edu.cn (P.H.); majunt@bjmu.edu.cn (J.M.); 2 Institute of Clinical Medical Sciences, China-Japan Friendship Hospital, Beijing 100029, China

**Keywords:** abnormal blood pressure, grip strength, BMI categories

## Abstract

**Background/Objectives**: The potential of relative grip strength as a biomarker for cardiovascular risk in college students is not well understood. **Methods**: Blood pressure status and grip strength metrics were analysed with multivariate logistic regression and restricted cubic spline models, utilising nationwide cross-sectional data from 42,591 Chinese college students. **Results:** After adjustment, higher absolute grip strength increased hypertension risk (High-level OR = 2.66; 95% CI: 2.21–3.20). In contrast, higher relative grip strength not only reduced risk overall (High-level OR = 0.41; 95% CI: 0.36–0.46) but also demonstrated consistent protective effects across all BMI subgroups (e.g., OR = 0.83 in overweight/obese individuals). **Conclusions**: Relative grip strength may present a valuable biomarker for cardiovascular risk assessment and the easy identification of at-risk individuals in all BMI categories.

## 1. Introduction

Hypertension is a key contributor to cardiovascular disease risk and has long been associated with the middle-aged and elderly population [1,2]. However, new epidemiological evidence indicates an alarming shifting trend, with the fastest growth moving towards the young population [3,4]. This warrants special attention, as an abnormal blood pressure in adolescence is a highly sensitive predictor of the long-term cardiovascular risk and disease burden [5,6]. College students comprise a major subpopulation among this high-risk youth group [7,8]. As future professionals and a key subject of national health programmes, their health bears profound social implications. However, current research on the youth population tends to focus on clinical hypertension, without sufficient attention to the much larger high-risk population falling into the high normal blood pressure category. National-level data reconstructing the actual distribution of abnormal blood pressure in the youth, as opposed to the aforementioned hypertension subpopulation, are also severely lacking, with most reports based on regional surveys [5,6,9].

Early risk stratification with simple yet effective biomarkers may be helpful. In particular, emerging data suggest that grip strength, a surrogate measure for general muscular fitness, is associated with cardiovascular health [10,11,12,13,14]. The college years (ages 18–22) represent a critical window for strength development, which may consequently serve as a pivotal period for potentially reducing cardiovascular risk [15,16]. Empirical evidence supporting the association between grip strength and blood pressure status, however, is not consistent [16,17,18,19]. Following a more in-depth analysis, this inconsistency may be attributed to a key methodological flaw. Past reports mainly assessed grip strength based on absolute measures, which are greatly confounded by body size (weight, BMI), without sufficiently controlling this variable as a confounder [20]. Relative grip strength (grip strength/weight or grip strength/BMI) may serve as a more meaningful biomarker, as it is more closely related to muscle mass than absolute grip strength. A critical evidence gap thus remains. Specifically, the abnormal blood pressure–grip strength association in the context of the BMI factor has yet to be established, and whether relative grip strength, the superior muscular strength measure that factors out the BMI confounder, displays a differential association with abnormal blood pressure status in different BMI categories remains completely unclear. Yet clarifying these associations is an essential first step toward evaluating the potential of muscle strength as an aberrant blood pressure risk screening tool.

To fill this critical gap, this study is designed as a nationwide cross-sectional study to (1) describe the national prevalence and regional variations in high normal blood pressure/hypertension among Chinese college students; (2) delineate the associations of absolute and relative grip strength with abnormal blood pressure status; and (3) specifically test whether and how such associations are altered in different BMI (under/normal weight versus overweight/obese) categories, thus laying the groundwork for future longitudinal studies and intervention trials [21]. It should be acknowledged that the cross-sectional design of the current study cannot infer causality with respect to the directionality of the identified associations (i.e., low muscle strength may precede or follow elevated blood pressure). Rather, the central purpose is to establish strong associations and interaction effects, which will inspire hypotheses for future longitudinal and interventional studies.

## 2. Materials and Methods

### 2.1. Subjects

The present study is a cross-sectional analysis of the 2019 Chinese National Survey on Students’ Constitution and Health (CNSSCH) [22]. The CNSSCH was the largest and most comprehensive national monitoring programme for the assessment of students’ physical fitness and individual health status in China. The sampling protocol was as follows: first, provinces were divided into high, medium, and low socioeconomic strata, and one city was randomly selected in each stratum; second, universities were randomly selected in each city, with a sample size ≥ 100 individuals for each grade from freshman to senior year. Accordingly, a balanced gender and urban–rural distribution of the sampled subjects was achieved. This study only included Han Chinese students, thereby excluding ethnic minorities and individuals who had missing data from the questionnaires, achieving both methodological validity and national representation of the obtained data [23]. All participants self-reported as being Han Chinese. This limitation was imposed to minimise heterogeneity and potential confounding by the broad ethnic differences in genetic and cultural factors.

### 2.2. Procedures

All measurements were conducted according to standardised protocols. Height and weight were measured to the nearest 0.01 m and 0.1 kg, respectively, using a portable stadiometer and digital scale (Model ZDR-P100-ST-01, Nanjing Zhidirui Information Technology Co., Ltd., Nanjing, China).Body mass index (BMI) was calculated as weight (kg) divided by height squared (kg/m^2^). We performed Chinese standards as part of our sensitivity analyses, with overweight and obesity (OWOB) defined as BMI ≥ 24 kg/m^2^ [24,25]. The Chinese BMI criteria were used because evidence suggested that for a given BMI, Chinese populations had higher adiposity-related health risk than Caucasian populations [24,25]. The use of ethnicity-appropriate cut-offs enhances the accuracy of risk stratification in this cohort [24,25]. Grip strength was assessed using an electronic dynamometer (Model LK-E1301, Lekang Instrument Co., Ltd., Changzhou, China). According to the corresponding protocols for each subject (2–3 trials), the maximum value was used.The present study used absolute grip strength (unit: kg) and relative grip strength (defined as the ratio of absolute grip strength to body weight, unit: kg/kg) as the main indicators. Both absolute grip strength and relative grip strength were divided into low, medium, and high according to the tertiles for hierarchical analysis. Blood pressure was measured according to the recommendations of the National High Blood Pressure Education Program Working Group in college students. Trained field staff measured blood pressure using an electronic sphygmomanometer (Model U720, Omron Corporation, Kyoto, Japan) following the standard auscultatory method. Prior to blood pressure measurement, the participants were seated and rested for a minimum of 10 min. An appropriate cuff size was selected based on arm circumference. The inflatable bladder within the cuff had a width of at least 40% of the arm circumference (measured midway between the olecranon and the acromion) and a length that covers 80–100% of the arm circumference. Such an approach allowed for the proper measurement of blood pressure among those with a large arm circumference. The cuff was placed on the right upper arm, positioned at heart level and approximately 2 cm above the antecubital fossa. Participants were seated with their feet flat on the floor and their arms supported at heart level. Systolic blood pressure and diastolic blood pressure were defined according to the first (K1) and fifth (K5) Korotkoff sounds, respectively. Three consecutive measurements were performed during a single visit, and the mean of the three measurements was used for all analyses. The classification of blood pressure was conducted in accordance with the national guideline [26,27]: normal blood pressure (systolic < 120 mmHg and diastolic < 80 mmHg), high normal blood pressure (systolic 120–139 mmHg or diastolic 80–89 mmHg), and hypertension (systolic ≥ 140 mmHg or diastolic ≥ 90 mmHg).

### 2.3. Statistical Analyses

All statistical analyses were performed using R Studio software (version 4.2.1; R Foundation for Statistical Computing, Vienna, Austria. URL: https://www.R-project.org/; accessed on 1 February 2025). Continuous variables were expressed as the mean ± SD, and categorical variables were compared by an ANOVA and χ 2-test. For logistic regression analysis, absolute and relative grip strength indices were standardised to Z-scores on the basis of sex and age. Quantile regression and logistic regression were used to evaluate the relationships between different grip strength measures (absolute and relative grip strength) and different blood pressure distributions. The estimated associations were derived by fitting logistic regression models using a restricted cubic spline (RCS). Linear combinations of the coefficients of the fitted models were computed to obtain the predicted probabilities that were plotted as a visual representation of these dose–response associations. All inferential analyses were two-tailed with a significance level of α = 0.05, and comprehensive model diagnostics were performed. The regression and RCS models were adjusted for the following covariates: area (urban/rural), age, sex, daily breakfast consumption frequency, egg consumption frequency, and dairy consumption frequency. A structured questionnaire was used to collect data on dietary habits. The frequency of breakfast consumption per day was used as a main behavioural metric [28,29]. In addition, due to their high importance and utility in the local breakfast context and the focus on nutrient intake, the frequency of eating eggs and dairy products as indicator food items was also separately analysed [30,31].

## 3. Results

### 3.1. Baseline Characteristics of the Study Population and Association Between Grip Strength and Blood Pressure Categories

Table 1 summarises the demographic and baseline characteristics of the study subjects. Among the 42,591 college students in the analysis, 23,587 (55.4%) had normal blood pressure, 16,755 (39.3%) had high normal blood pressure, and 2249 (5.3%) had hypertension. Among the 42,591 college students, 34,352 (80.7%) were of underweight and normal weight (NLW), and 8239 (19.3%) were in the OWOB group. Age groups: 19 years old—10,790 (25.3%); 20 years old—11,060 (26.0%); 21 years old—10,940 (25.7%); 22 years old—9801 (23.0%). In terms of gender, there were 21,174 males (49.7%) and 21,417 females (50.3%). There were 21,456 (50.4%) urban and 21,135 (49.6%) rural students.

Table 2 shows that compared with the normal blood pressure group, height, weight, BMI, and absolute grip strength were significantly higher in the subgroups (including BMI, age, gender, and urban–rural populations) of high normal blood pressure and hypertension (*p* < 0.05). In addition, in all subgroups, the absolute grip strength of the hypertension patients was significantly higher than that of the high normal blood pressure patients (*p* < 0.05). Relative grip strength was also significantly higher in the subgroups of different BMI, age, and urban–rural populations. However, in the subgroups of gender, both the high normal blood pressure and hypertension groups exhibited significantly lower relative grip strength than the corresponding normal blood pressure groups. In addition, there were significant differences in relative grip strength between males and females in the subgroups of high normal blood pressure and hypertension (*p* < 0.05).

### 3.2. Regional Distribution of Blood Pressure Abnormalities and Grip Strength Levels in College Students in Different Chinese Provinces

**Figure 1:**
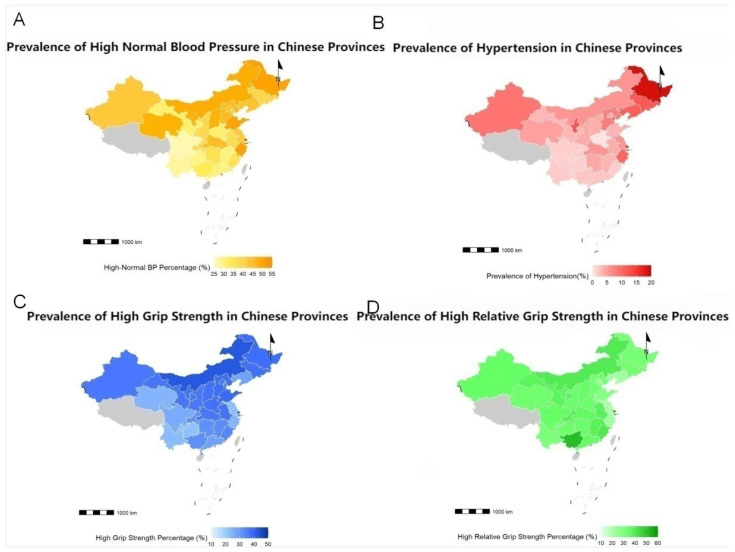
Abnormal blood pressure status and grip strength in individuals aged 19–22 across provinces in China. (**A**) High normal blood pressure. (**B**) Hypertension. (**C**) High absolute grip strength. (**D**) High relative grip strength.

Figure 1 shows the distribution of the prevalence of high normal blood pressure and hypertension, as well as the proportion of different grip strength indicators, in Chinese college students across different provinces. The largest proportion of college students with high normal blood pressure was found in Zhejiang (51.8%), Shandong (51.3%), and Heilongjiang (51.1%). The highest proportion of college students with hypertension was observed in Heilongjiang (19.0%), Ningxia (12.3%), and Jilin (11.7%). Inner Mongolia (42.7%), Shandong (39.3%), and Hebei (39.0%) had the largest proportion of college students with high absolute grip strength, while the largest proportion of college students with high relative grip strength indicators was observed in Guangxi (51.6%), Fujian (40.4%), and Inner Mongolia (39.9%). These findings indicate large regional differences in cardiovascular risk factors, implying that regional characteristics may be associated with health status.

### 3.3. Association Between Grip Strength Indicators (Absolute and Relative Grip Strength) and Blood Pressure Abnormalities

**Figure 2:**
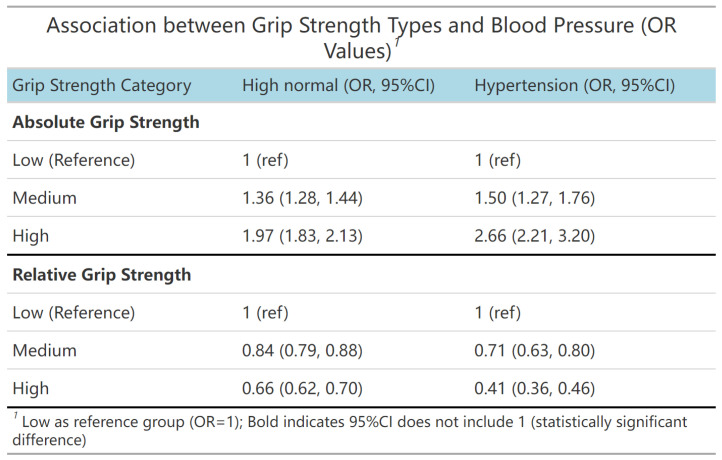
Independent associations of absolute and relative grip strength (categorised into low, medium, and high groups) with abnormal blood pressure status, using low grip strength group as reference. Note: Adjusted for residence area, age, sex, daily breakfast frequency, egg consumption, and dairy consumption. Abbreviation: Ref. = reference; OR = odds ratio; CI = confidence interval.

Figure 2 demonstrates a significant association between grip strength and blood pressure status. After adjusting for covariates, compared with the low absolute grip strength group, higher absolute grip strength was associated with an increased risk of high normal blood pressure (Moderate level: OR = 1.36, 95% CI: 1.28–1.44; High level: OR = 1.97, 95% CI: 1.83–2.13) and hypertension (Moderate absolute grip strength level: OR = 1.50, 95% CI: 1.27–1.76; High absolute grip strength level: OR = 2.66, 95% CI: 2.21–3.20). Compared with the low relative grip strength group, higher relative grip strength was associated with a reduced risk of high normal blood pressure (Moderate level: OR = 0.84, 95% CI: 0.79–0.88; High level: OR = 0.66, 95% CI: 0.62–0.70) and hypertension (Moderate level: OR = 0.71, 95% CI: 0.63–0.80; High level: OR = 0.41, 95% CI: 0.36–0.46).

**Figure 3:**
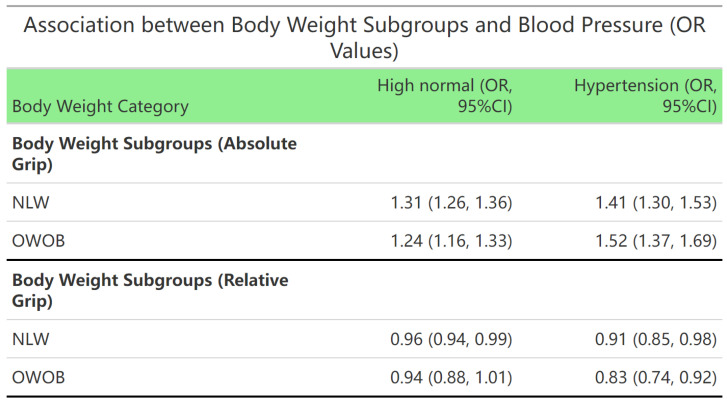
Evaluation of whether the associations differ across BMI categories (underweight/normal weight vs. overweight/obese). Note: Adjusted for residence area, age, sex, daily breakfast frequency, egg consumption, and dairy consumption. Abbreviation: Ref. = reference; OR = odds ratio; CI = confidence interval.

Within BMI status subgroups, each standard deviation increase in absolute grip strength was associated with higher risks of high normal blood pressure (NLW group: OR = 1.31, 95% CI: 1.26–1.36; OWOB group: OR = 1.24, 95% CI: 1.16–1.33) and hypertension (NLW group: OR = 1.41, 95% CI: 1.30–1.53; OWOB group: OR = 1.52, 95% CI: 1.37–1.69). Concurrently, each standard deviation increase in relative grip strength was associated with reduced incidence risk for both high normal blood pressure (NLW group: OR = 0.96, 95% CI: 0.94–0.99) and hypertension (NLW group: OR = 0.91, 95% CI: 0.85–0.98; OWOB: OR = 0.83, 95% CI: 0.74–0.92). These findings suggest that relative grip strength may be a more suitable indicator for cardiovascular risk assessment than absolute grip strength (Figure 3).

**Figure 4:**
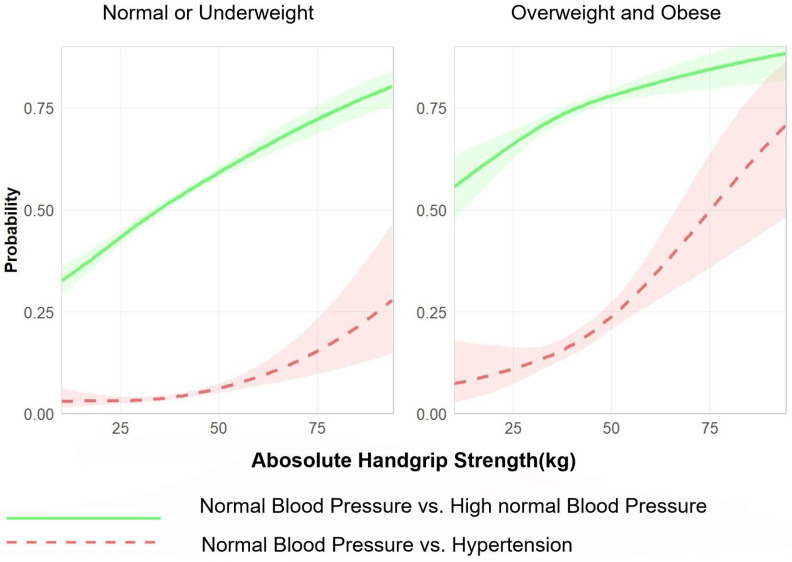
Independent associations of absolute grip strength with abnormal blood pressure status, stratified by BMI group (underweight/normal weight vs. overweight/obese). Note: Adjusted for residence area, age, sex, daily breakfast frequency, egg consumption, and dairy consumption.

**Figure 5:**
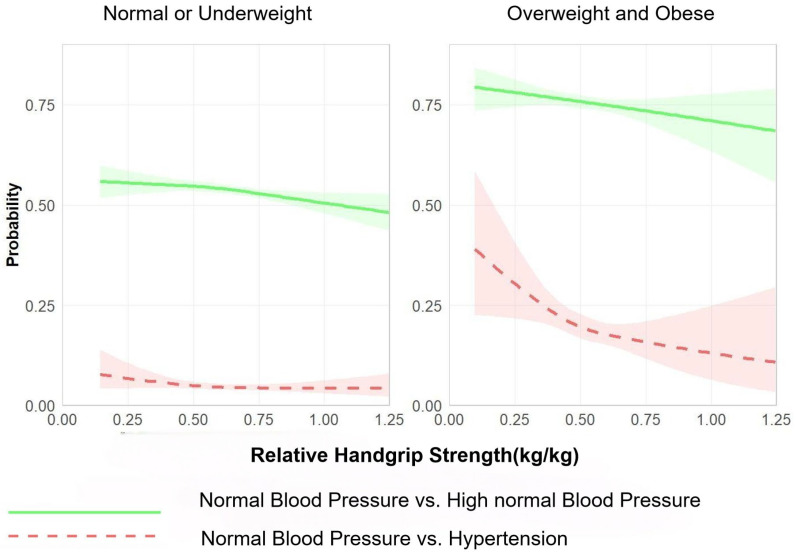
Independent associations of relative grip strength with abnormal blood pressure status, stratified by BMI group (underweight/normal weight vs. overweight/obese). Note: Adjusted for residence area, age, sex, daily breakfast frequency, egg consumption, and dairy consumption.

Figure 4 and Figure 5 show estimated curves of the probability of a positive outcome according to the value of continuous predictors, as derived from the generalised linear models using restricted cubic splines stratified by BMI group (normal or underweight vs. overweight or obese). The analyses were based on comparisons at two different levels: (1) normal blood pressure and high normal blood pressure and (2) normal blood pressure and hypertension. Absolute grip strength showed a positive dose–response relationship with the prevalence of high normal blood pressure and hypertension. Notably, this association was more pronounced among OWOB students than their NLW counterparts (Figure 4). Conversely, relative grip strength exhibited a negative dose–response relationship with the prevalence of high normal blood pressure and hypertension. This inverse correlation was also more evident in OWOB students than in NLW students (Figure 5).

## 4. Discussion

As this is one of the few nationwide studies to assess relative grip strength in Chinese university students, we propose that relative grip strength could be a biomarker for blood pressure health. Specifically, higher relative grip strength was associated with a lower prevalence of both high normal blood pressure and hypertension in all BMI categories, most strongly among overweight/obese individuals. This relationship is particularly noteworthy given the high prevalence of high normal blood pressure in this young population—a condition linked to adverse cardiovascular changes even in the absence of clinical hypertension [32,33,34]. Relative grip strength may therefore not only serve as a marker of muscular functioning but as a more integrative marker that could be harnessed as an easy and cost-effective tool to facilitate early risk stratification, especially in settings with limited resources [35].

A major strength of this study is its investigation into the inconsistent grip strength–hypertension association in the previous literature by distinguishing between absolute and relative grip strength [17,36,37,38]. We show that while absolute grip strength revealed positive associations with blood pressure in all BMI groups and was thus useless in prediction [39], relative grip strength consistently captured a strong inverse association. This is consistent with emerging evidence that weight-adjusted strength is a stronger predictor of overall cardiometabolic risk [40,41,42]. The physiologic basis of this relationship lies in the integrative nature of relative grip strength. It represents an individual’s neuromuscular capacity scaled to their metabolic load. With weight gain, absolute grip strength increases to some extent but typically with an accumulation of fat infiltration, decreased muscle quality, and reduced overall power, meaning less strength per unit of body mass. In addition, because of the direct bearing of body weight on blood volume and cardiac load, which regulates metabolic homeostasis, it helps contribute directly to hypertension through other mechanisms related to excessive sympathetic activity and insulin resistance [43,44]. It thus better reflects the delicate interplay between functional reserve and cardiovascular–metabolic compensation while inherently adjusting for the weight-driven hemodynamic and metabolic perturbations that drive hypertension. Relative grip strength thus demonstrates key potential as a biomarker that can be harnessed to identify at-risk individuals [45] while suggesting that interventions should target both muscle strengthening and weight normalisation.

In the NLW and OWOB groups, higher absolute grip strength is associated with increased risks of high normal blood pressure/hypertension, while higher relative grip strength is associated with decreased risks of high normal blood pressure/hypertension at the higher absolute grip strength levels. Previous studies are consistent in that muscle strength is the gold standard for predicting health outcomes [46,47,48,49,50]. In addition to its association with blood pressure, higher muscle strength is associated with a lower risk of chronic diseases related to metabolic abnormalities, such as metabolic syndrome, diabetes, and obesity [42,51,52]. Moreover, other studies have found an association of grip strength with the risk of chronic diseases. However, this finding contrasts with a previous study in children and adolescents, which reported that grip strength was positively associated with hypertension risk only in obese individuals but not in those with normal weight [24]. Our study results indicate that the association between absolute/relative grip strength and hypertension is independent of BMI status. For each standard deviation increase in absolute grip strength, the risk of high normal blood pressure/hypertension increases more in OWOB individuals than in NLW individuals, while the risk decreases more for each standard deviation increase in relative grip strength. One possible explanation for this interaction is that OWOB individuals have more significant insulin resistance or an inflammatory status, and within this population, higher muscle strength is associated with greater metabolic benefits. In addition, increased grip strength is also accompanied by increased physical activity and quality of life. Higher muscle strength has also been associated with broader health benefits in OWOB individuals [53].

The limitations of this study include the following: First and foremost, the cross-sectional design precludes causal inference. Specifically, we cannot determine whether lower relative muscle strength precedes or results from elevated blood pressure, nor can we assess the longitudinal trajectories of these associations. Second, Han Chinese students represented our entire sample. Although this was necessary to control for major ethnic confounding and to obtain a first estimate of the associations that is as “clean” as possible, it also has important limitations: Due to the single-ethnicity composition of our study sample, we squandered a rare opportunity to probe deeply into the mechanisms of gene–environment interactions, which is increasingly considered an epicentre of scientific questions in understanding the innate population differences in complex phenotypes such as blood pressure regulation. Specifically, within the context of this study, we are fundamentally unable to address the following key mechanistic questions. The environmental modification of genetic effects: Is the grip strength–blood pressure association identified in Han Chinese students observed in this study further modified by certain environmental exposure, such as dietary patterns, physical activity patterns/types, or climatic adaptation? If so, would the moderation pattern found in Han Chinese people also apply to other ethnic groups, given their vastly different genetic backgrounds? The genetic susceptibility to environmental exposure: Do people with different genetic backgrounds differ in their blood pressure sensitivity to muscle strength changes? The protective association characterised in Han Chinese people might be stronger or weaker in people from other ethnic backgrounds due to the presence or absence of certain ethnicity-specific genetic profiles. Gene–culture coevolutionary pathways: Have long-standing cultural practices and lifestyles resulted in certain ethnic-specific traits/mutations in muscle metabolism and cardiovascular adaptation because of certain genetic selection or epigenetic mechanisms? Our study design is unfortunately not suited to address this deep evolutionary biology question. The lack of investigation at this level of mechanism not only limits our understanding at the deepest level of biological foundation but also, more importantly, limits the translation of the research findings into precision public health practices. Third, the dietary assessment was based on a frequency questionnaire rather than a quantitative questionnaire. Thus, the nutrient composition (for example, fat, calories, and specific micronutrients) of breakfast, which might provide additional mechanistic insights, could not be analysed, and the consumption frequencies of key food groups (eggs, dairy) were used as proxies. Additionally, the absence of biological markers (e.g., inflammatory indices, insulin levels) and objective measures of physical activity limits the mechanistic interpretation of the observed associations. Furthermore, the categorisation of handgrip strength into tertiles may obscure potential non-linear effects at the extremes of the distribution. An essential selection bias in this study should be noted. The sample is exclusively drawn from the CNSSCH, and the CNSSCH’s sampling framework automatically excludes young adults not in higher education. This comprises young adults not in higher education who did not enter higher education or never even entered the education system or dropped out early as a result of health (long-term illness, disabilities, protective against population-representative sampling, etc.) or socioeconomic (family poverty, early work needs) reasons. Constrained by the widely recognised social gradient between education and health, young adults not in higher education may have poorer health behaviours, more constrained healthcare access, unhealthier constitutional backgrounds, and poorer health outcomes than college students overall. The prevalence of outcomes with negative health determinants among young adults not in higher education should therefore be much higher than that of college students. The estimated prevalence of high normal blood pressure and hypertension in this study, based solely on Chinese college students, is likely to be substantially lower than the true prevalence in the overall Chinese youth population of the same age group, including those with known or unknown cardiometabolic risks excluded from or that exited from the education system. More importantly, this “healthy population bias” might lead to an underestimate of the association between key predictors. For example, the protective association between grip strength and blood pressure may exist in a more pronounced or different pattern among non-student populations with higher health risks. Because this study did not include these potentially less healthy populations (with a fuller spectrum of risk), the estimated effect size (e.g., odds ratios) is likely to be diluted and restricts the generalisability to Chinese youth as a whole. To address the limitations of ethnic homogeneity and potential selection bias, future studies should aim to include more diverse populations, encompassing various ethnicities and socioeconomic backgrounds, to validate our findings and explore the role of genetic–environmental interactions. Additional research that incorporates comprehensive dietary records and biological markers is necessary in the future to evaluate the outcomes between precise breakfast nutrient intake and health consequences.

## 5. Conclusions

Moreover, this study suggests that synergistic attention between muscle strength and body weight control may be relevant for promoting blood pressure health (as opposed to focusing solely on enhancing muscle strength). Relative grip strength is an important protective factor for hypertension in all BMI status groups, especially for OWOB college students.

## Figures and Tables

**Table 1:** The descriptive characteristics of the participants included in this study.

Variable		Basic Blood Pressure Categories		
	Total	Normal Blood Pressure	High Normal Blood Pressure	Hypertension
		(*n* = 23,587)	(*n* = 16,755)	(*n* = 2249)
**Age Groups, *n* (%)**				
19	10,790	6378 (59.1)	3934 (36.5) *	478 (4.4) *
20	11,060	6227 (56.3)	4244 (38.4) *	589 (5.3) *
21	10,940	5849 (53.5)	4497 (41.1) *	594 (5.4) *
22	9801	5133 (52.4)	4080 (41.6) *	588 (6.0) *
**Sex,** ***n*** **(%)**				
Male	21,174	8216 (38.8)	11,199 (52.9) *	1759 (8.3) *
Female	21,417	15,371 (71.8)	5556 (25.9) *	490 (2.3) *
**Areas,** ***n*** **(%)**				
Urban	21,456	11,785 (54.9)	8508 (39.7) *	1163 (5.4)
Rural	21,135	11,802 (55.8)	8247 (39.0) *	1086 (5.1)
**Handgrip Strength,** ***n*** **(%)**				
Low	14,238	10,433 (73.3)	3500 (24.6) *	305 (2.1)
Moderate	14,180	8098 (57.1)	5437 (38.3) *	645 (4.5)
High	14,173	5056 (35.7)	7818 (55.2) *	1299 (9.2)
**Relative Grip Strength, *n* (%)**				
Low	14,200	8818 (62.1)	4747 (33.4) *	635 (4.5) *
Moderate	14,196	8017 (56.5)	5401 (38.0) *	778 (5.5) *
High	14,195	3133 (44.4)	3504 (49.6) *	421 (6.0) *
**BMI Group, *n* (%)**				
NLW	34,352	20,902 (60.8)	12,159 (35.4) *	1291 (3.8) *
OWOB	8239	2685 (32.6)	4596 (55.8) *	958 (11.6) *

Note: a Data described by *n* (%). BMI, body mass index; NLW, normal or underweight; OWOB, overweight and obese; * means *p* < 0.05 compared to the normal blood pressure group.

**Table 2:** Basic characteristics, blood pressure, and handgrip strength in Chinese college students aged 19–22 years in the Chinese National Survey on Students’ Constitution.

Variable	Basic Blood Pressure Categories		
	Normal Blood Pressure	High Normal Blood Pressure	Hypertension
	(*n* = 23,587)	(*n* = 16,755)	(*n* = 2249)
Height (cm)	164.00 (8.26)	170.00 (8.30) *	172.00 (8.09) *
Weight (kg)	56.30 (9.96)	64.70 (13.00) *	71.50 (16.20) *
BMI	20.80 (2.82)	22.40 (3.70) *	24.00 (4.65) *
Handgrip Strength	30.70 (9.67)	37.20 (10.60) *	40.20 (10.80) *
Relative Grip Strength	0.55 (0.14)	0.58 (0.15) *	0.57 (0.15)
BMI			
Handgrip Strength			
NLW	30.30 (9.41)	36.00 (10.30) *	38.00 (10.60) *
OWOB	34.50 (10.80)	40.3 (10.6) *	43.10 (10.40) *
Relative Grip Strength			
NLW	0.56 (0.14)	0.61 (0.15) *	0.62 (0.15) *
OWOB	0.47 (0.13)	0.51 (0.13) *	0.51 (0.12) *
Age			
Handgrip Strength			
19	30.50 (9.66)	36.90 (10.40) *	40.00 (10.70) *
20	30.60 (9.42)	37.00 (10.70) *	40.00 (10.60) *
21	30.80 (9.75)	37.10 (10.40) *	40.60 (11.00) *
22	31.00 (9.88)	37.70 (10.80) *	40.10 (10.90) *
Relative Grip Strength			
19	0.54 (0.15)	0.57 (0.15) *	0.57 (0.15) *
20	0.54 (0.14)	0.58 (0.15) *	0.57 (0.15) *
21	0.55 (0.14)	0.58 (0.14) *	0.58 (0.14) *
22	0.55 (0.14)	0.59 (0.15) *	0.57 (0.15) *
Sex			
Handgrip Strength			
Male	40.60 (7.88) *	42.50 (8.09) *	43.8 (8.85) *
Female	25.40 (5.48) *	26.40 (5.77) *	27.1 (6.18) *
Relative Grip Strength			
Male	0.65 (0.14) *	0.63 (0.14) *	0.60 (0.14) *
Female	0.49 (0.11) *	0.48 (0.11) *	0.47 (0.12) *
Areas			
Handgrip Strength			
Urban	30.60 (9.73)	36.90 (10.50) *	39.90 (11.00) *
Rural	30.90 (9.60)	37.40 (10.70) *	40.40 (10.70) *
Relative Grip Strength			
Urban	0.53 (0.14)	0.57 (0.14) *	0.56 (0.15) *
Rural	0.56 (0.14)	0.60 (0.15) *	0.59 (0.15) *

Note: Data described by mean ± SD. * means *p* < 0.05 compared to normal blood pressure group.

## Data Availability

The data presented in this study are available on request from the corresponding author due to privacy and ethical considerations.

## Abbreviations

The following abbreviations are used in this manuscript:

BMI	Body mass index
OWOB	Overweight and obese
NLW	Underweight and normal weight
RCS	Restricted cubic spline
